# LAMA4 upregulation is associated with high liver metastasis potential and poor survival outcome of Pancreatic Cancer

**DOI:** 10.7150/thno.47001

**Published:** 2020-08-13

**Authors:** Biao Zheng, Jianhua Qu, Kenoki Ohuchida, Haimin Feng, Stephen Jun Fei Chong, Zilong Yan, Yicui Piao, Peng Liu, Nan Sheng, Daiki Eguchi, Takao Ohtsuka, Kazuhiro Mizumoto, Zhong Liu, Shazib Pervaiz, Peng Gong, Masafumi Nakamura

**Affiliations:** 1 Department of General Surgery, Shenzhen University General Hospital / Shenzhen University Clinical Medical Academy, Shenzhen, Guangdong 518055, China.; 2 Department of Surgery and Oncology, Graduate School of Medical Sciences, Kyushu University, Fukuoka 812-8582, Japan.; 3 Department of Physiology, Yong Loo Lin School of Medicine, National University of Singapore (NUS), Singapore 117593.; 4 Advanced Medical Initiatives, Graduate School of Medical Sciences, Kyushu University, Fukuoka 812-8582, Japan.; 5 Hepato-pancreato-biliary Surgery Department, Peking University Shenzhen Hospital, Shenzhen, Guangdong 518055, China.; 6 Department of Critical Care Medicine, National Cancer Center/Cancer Hospital & Shenzhen Hospital, Chinese Academy of Medical Sciences and Peking Union Medical College, Shenzhen, Guangdong 518116, China.; 7 Cancer Center of Kyushu University Hospital, Fukuoka 812-8582, Japan.; 8 NUS Graduate School for Integrative Sciences and Engineering, National University of Singapore, Singapore 117593.; 9 National University Cancer Institute, National University Health System, Singapore 119074.; 10 Carson International Cancer Research Centre, Shenzhen University School of Medicine, Shenzhen, Guangdong 518055, China.

**Keywords:** LAMA4, metastasis, cancer-associated fibroblasts, tumor severity, pancreatic cancer

## Abstract

**Rationale:** Pancreatic cancer is one of the most difficult cancers to manage and its poor prognosis stems from the lack of a reliable early disease biomarker coupled with its highly metastatic potential. Liver metastasis accounts for the high mortality rate in pancreatic cancer. Therefore, a better understanding of the mechanism(s) underlying the acquisition of the metastatic potential in pancreatic cancer is highly desirable.

**Methods:** Microarray analysis in wild-type and highly liver metastatic human pancreatic cancer cell lines was performed to identify gene expression signatures that underlie the metastatic process. We validated our findings in patient samples, nude mice, cell lines and database analysis.

**Results:** We identified a metastasis-related gene, laminin subunit alpha 4 (*LAMA4*), that was upregulated in highly liver metastatic human pancreatic cancer cell lines. Downregulation of LAMA4 reduced the liver metastatic ability of pancreatic cancer cells *in vivo*. Furthermore, LAMA4 expression was positively correlated with tumor severity and *in silico* analyses revealed that LAMA4 was associated with altered tumor microenvironment. In particular, our *in vitro* and i*n vivo* results showed that LAMA4 expression was highly correlated with cancer-associated fibroblasts (CAFs) level which may contribute to pancreatic cancer metastasis. We further found that LAMA4 had a positive effect on the recruitment and activity of CAFs.

**Conclusions:** These data provide evidence for LAMA4 as a possible biomarker of disease progression and poor prognosis in pancreatic cancer. Our findings indicate that LAMA4 may contribute to pancreatic cancer metastasis via recruitment or activation of CAFs.

## Introduction

Pancreatic cancer is one of the deadliest and most aggressive forms of cancer. The relatively rapid progression and high metastatic potential of pancreatic cancer account for the high mortality rate 1. Liver metastasis is considered the most significant problem in pancreatic cancer, as the majority of patient deaths are from pancreatic cancer liver metastasis 2-4. Therefore, identification of specific signaling networks or factors that are predictive of early disease and/or metastatic potential could be instrumental in designing target-specific strategies for pancreatic cancer patients.

The tumor microenvironment (TME) is a milieu that dictates the interactions between the tumor and the extracellular matrix as well as stromal cells and immune cells 5. Cancer-associated fibroblasts (CAFs) are the major component of stromal cells in the TME of pancreatic cancer. CAFs promote the proliferation and growth of pancreatic cancer cells and thus contribute to the progression, invasion and metastasis of pancreatic tumors 6-8. A deeper understanding of the tumor-CAFs interaction may identify potential therapeutic strategies for preventing metastasis and progression of pancreatic cancer.

Laminins are a family of extracellular glycoproteins that play important roles in providing a microenvironment for cell functions 9 through their regulation of cell differentiation, migration and adhesion 10, 11. Laminin subunit alpha 4 (LAMA4) belongs to the laminin family. LAMA4 is mainly distributed in endothelial basement membranes and mesenchymal origin 12-16. LAMA4 plays a significant role in promoting survival, proliferation and migration in various cell types 17. Previous studies also showed that LAMA4 overexpression is associated with poor survival, metastasis and migration in renal cell carcinoma 16, 18. Furthermore, high expression of LAMA4 has been associated with invasiveness in breast cancer 19. However, the function and molecular mechanisms of LAMA4 in pancreatic cancer have not been systematically studied.

In this article, we describe the association between LAMA4 upregulation and metastatic potential of pancreatic cells using established cell lines, murine models as well as patient derived clinical material. Notably, downregulation of LAMA4 inhibited liver metastasis in a murine model of pancreatic cancer. Furthermore, tumor-derived LAMA4 was highly positively correlated with recruitment of CAFs and had a positive effect on viability and migration of CAFs in pancreatic cancer. Thus, we suggest that LAMA4-mediated recruitment or activation of CAFs may contribute to the metastatic potential of pancreatic cancer.

## Material and Methods

### Establishment of highly liver metastatic human pancreatic cancer cell lines

The establishment of highly liver-metastatic human pancreatic cancer cell lines was performed as described in our previous study 20. 4-weeks-old BALB/c AJcl nu/nu female mice (CLEA, Tokyo, Japan) were used as the xenograft model. We injected PANC-1 (1 × 10^6^) cells into pancreas of nude mice. Hepatic metastases were harvested at 6-10 weeks after injection. Primary culture was carried out by using harvested tissue with collagenase. We then injected the cultured cells into the tail of pancreas of nude mice. We repeated same process five rounds to establish the highly liver-metastatic PANC-1 cell line (Figure S1A).

### Microarray analysis

Microarray analyses were performed using wild-type (WT) and highly liver metastatic (HM) SUIT-2 and PANC-1 cells. The Human WG-6 Expression BeadChip was used for analyses. We analyzed the datasets by using Illumina BeadStudio program. The datasets were uploaded to the GEO database (GEO accession: GSE144909).

### Pancreatic tissue preparation

We obtained the pancreatic cancer tissues from 140 patients at the Department of Surgery and Oncology, Graduate School of Medical Sciences, Kyushu University, Fukoka, Japan. Normal pancreatic tissues from resected pancreases (from patients with bile duct cancer) were used as the controls. We summarized all clinicopathological parameters of 140 patients in Table S1. Our research was approved by the Ethics Committee of Kyushu University (24-222, 25-117).

### Immunohistochemistry

Immunohistochemistry (IHC) staining was performed in tissue sections as described previously 21. Sections were incubated with anti-LAMA4 antibody (ab242198, Abcam) or anti-α-SMA antibody (ab32575, Abcam) at 4°C for 12h. We identified carcinoma cells morphology at 200× magnification. ImageJ software was used for quantitative analysis of LAMA4 expression, and DAB and hematoxylin staining were digitally separated using an ImageJ plugin for color deconvolution (Figure S1B). The deconvoluted image was subjected to histogram analysis. For each section, three fields were randomly selected at 200× magnification and the amount of DAB signal intensity was measured; these data were converted into optical density (OD) units using ImageJ software, and positive staining was adjusted by subtracting background control signals. The average value of all three fields was used to assign a staining value to each section. The results were recorded as average OD value/area. LAMA4 staining of blood vessel was used as a positive control. The average OD value (0.129486) of LAMA4 expression on blood vessels was used as a threshold. Patients were then assigned into LAMA4 high and LAMA4 low expression groups according to this threshold (Figure S1C). Cells with a spindle-shaped morphology that positively stained for α-SMA were defined as CAFs-like cells and CAFs with α-SMA expression were distinguished from blood vessels by CD31 staining (ab28364, Abcam). The α-SMA-positive areas around tumor cells were evaluated in three random fields for each section by ImageJ. The relative level of CAFs was calculated as percentage of α-SMA-positive staining area in each section.

### Cell culture and treatment

Pancreatic cancer cell lines used in this study were purchased as follows: PANC-1 (RIKEN BioResource Center, Ibaraki, Japan), SUIT-2, MIA-PaCa-2 (Japanese Cancer Resource Bank, Tokyo, Japan), AsPC-1, CFPAC-1, and Capan-2 lines (American Type Culture Collection, Manassas, VA, USA). The primary cultured CAFs were established as described in previous study 21, 22. The CAFs were α-SMA, CD90, Vimentin, GFAP and Nestin positive, and CK19 negative 21. CAFs of passage 3-8 were used in experiments. Cells were maintained in Dulbecco's modified Eagle medium (DMEM) supplemented with 10% fetal bovine serum. The human pancreatic ductal epithelial (HPDE) cell line was from the lab of Dr. Ming-Sound Tsao (Toronto General Hospital/Research Institute, University of Toronto, Canada). HPDE cells were maintained in HuMedia-KG2 (KURABO, Osaka, Japan) medium. We obtained the Human umbilical vein endothelial cells (HUVECs) from Lonza (C2517A) which was cultured in EBM-2 medium. To collect conditioned medium, SIUT-2 cells were cultured in 5 mL DMEM containing 10% FBS. The medium was collected 24-48 h later and centrifuged at 1500 rpm for 5 min. Supernatants were collected and stored at 4°C until used. Fresh DMEM containing 10% FBS was used as control. Recombinant Human Laminin α4 (2.0 μg/mL, 7340-A4, R&D Systems) and Human Laminin α4 Antibody (5 μg/mL, AF7340, R&D Systems) were reconstituted following the manufacturer's recommendations and used at the indicated doses.

### shRNA and luciferase plasmid transfection

Two *LAMA4* shRNA vectors (V2LHS_133879/363651, Thermo) and a firefly luciferase vector (#LVP326, GenTarget) were amplified and transfected into SUIT-2 and AsPC-1 cells. *LAMA4* shRNA clones and luciferase clones were selected by Puromycin (#631305, Takara) or blasticidin S hydrochloride (#15205, Sigma-Aldrich). Cells were cultured in selection media for 3 weeks. *LAMA4* downregulation was examined by qRT-PCR and western blot analyses. Cells transfected with non-targeting shRNA (V13111501, Thermo) was used as a control. D-Luciferin Potassium salt (150 μg/ml, #LK10000, OZBIOSCIENCES, Marseille, France) was used for Luciferase assays.

### qRT-PCR analysis

TRIzol RNA isolation reagent (ThermoFisher) was used for extraction of total RNA. *LAMA4* mRNA levels were assessed using qRT-PCR method with SYBR Premix EX Taq II (TaKaRa). The mRNA level of GAPDH was used as control. The following primer sets were used for amplification: *LAMA4* (forward primer) TTCGAACACCAGCTGACAAC and (reverse primer) AGGTAACCATTGCGCATTTC; *GAPDH* (forward primer) AGCCACATCGCTCAGACAC and (reverse primer) GCCCAATACGACCAAATCC 19.

### Western blot analysis

Mini-PROTEAN TGX Precast Gels (Bio-Rad Laboratories) was used for electrophoresis. Membranes were incubated at 4°C for 12h with anti-LAMA4 (ab242198, Abcam), anti-Glyceraldehyde 3-phosphate dehydrogenase (GAPDH) (ab8245, Abcam) and anti-β-actin antibodies (ab8227, Abcam). ChemiDoc XRS (Bio-Rad Laboratories) was used to detect signals of immunoblot.

### *In vivo* experiments

For orthotopic transplantation experiments using pancreatic cancer cells (1×10^6^) suspended in 50 μL DMEM were injected into the tail of the pancreas of nude mice. Mice were sacrificed 6-7 weeks after transplantation, and livers were resected and the hepatic metastases were evaluated. Metastatic tumors in liver were also validated by CT scan. For liver metastasis model experiments using WT or *LAMA4*-depleted luciferase-expressing SUIT-2 cells, WT or *LAMA4*-depleted luciferase-expressing AsPC-1 cells (n=5) were suspended in 50 μL DMEM and injected into the spleens, and spleens were resected 5 min after injection. We examined Luciferin emission every week using the IVIS Spectrum (Caliper Life Sciences) after injecting D-luciferin into the intraperitoneal cavity of nude mice. Quantification of emission was performed using Living Image software version 4.4.

### Cell viability assay of non-contact co-culture

Human pancreatic cancer cells were seeded (1 × 10^3^ cells/well) and cultured in 96-well plates and cell viability was assessed using the CellTiter-Glo Luminescent Cell Viability Assay Kit (G7570, Promega) following the manufacturer's instructions. Cell viability was examined by measuring the luminescent intensity at specified times using a microplate reader (Tecan, Männedorf, Switzerland). For co-cultures cell viability assay, CAFs were seeded (1 × 10^4^ cells/well) and cultured in the lower chambers. After 24h, the cancer cells were seeded (2 × 10^5^ cells/ml) and cultured in the upper transwell chamber (3 μm pore size). Values from wells only containing cell culture medium was used to evaluate the background.

### Cell migration and invasion assays

We assessed cancer cell migration ability and invasion ability by examining the number of cells migrating or invading through uncoated or Matrigel-coated transwell chambers (8 μm pore size) as previously described 23. For co-culture migration assay, cancer cells (2 × 10^5^ cells/mL) were seeded and cultured in the lower chambers, and CAFs (4 × 10^4^ cells/well) were seeded and cultured in the upper transwell chamber. We determined migration ability 24 h after cell seeding. The invasiveness was determined 48h after cell seeding. In both cell migration and invasion assays, migrated or invaded cells were fixed with 70% ethanol. Then the cells were stained with hematoxylin and eosin. We counted cells in 5 random fields (100 × magnification).

### Database-based bioinformatics data mining

ICGC (International Cancer Genome Consortium) gene expression data of pancreatic cancer plus clinical data were obtained from ICGC website. ICGC-CA was the pancreatic cancer dataset from the Canada Pancreatic Cancer Genome Initiative. ArrayExpress gene expression dataset and corresponding clinical information of pancreatic cancer were downloaded from the ArrayExpress database at EMBL-EBI 24 under accession number E-MTAB-6134. The expression datasets of normal pancreas and pancreatic tumors used for the analyses described in this study were generated by the GTEx Portal (The Genotype-Tissue Expression project) and TCGA database (The Cancer Genome Atlas Program). The datasets were arranged by UCSC Xena 25. The datasets were downloaded from UCSC Xena on 25 Sep 2019. The GTEx Project was supported by the Common Fund of the Office of the Director of the National Institutes of Health and by NCI, NHGRI, NHLBI, NIDA, NIMH, and NINDS. Bioinformatic analysis and statistical analysis were conducted using Perl language, R language and GraphPad prism.

### Statistical analysis

The chi-squared test was performed to assess relationships between LAMA4 protein expression and clinicopathological features. For results of *in vitro* experiments, values are expressed as the mean ± SEM. Comparisons of RT-QPCR were carried out using the Student's t-test. Comparisons of RNA expression in bioinformatic analysis were performed using the Wilcox test and Kruskal-Wallis test. Kaplan-Meier analyses were compared using the log-rank test. Spearman's correlation test was performed to examine the correlation coefficients in the study. *P* < 0.05 was used to define statistical significance. Statistical analysis was conducted using R language and GraphPad prism.

## Results

### LAMA4 is upregulated in highly liver metastatic human pancreatic cancer cell lines

**Figure 1:**
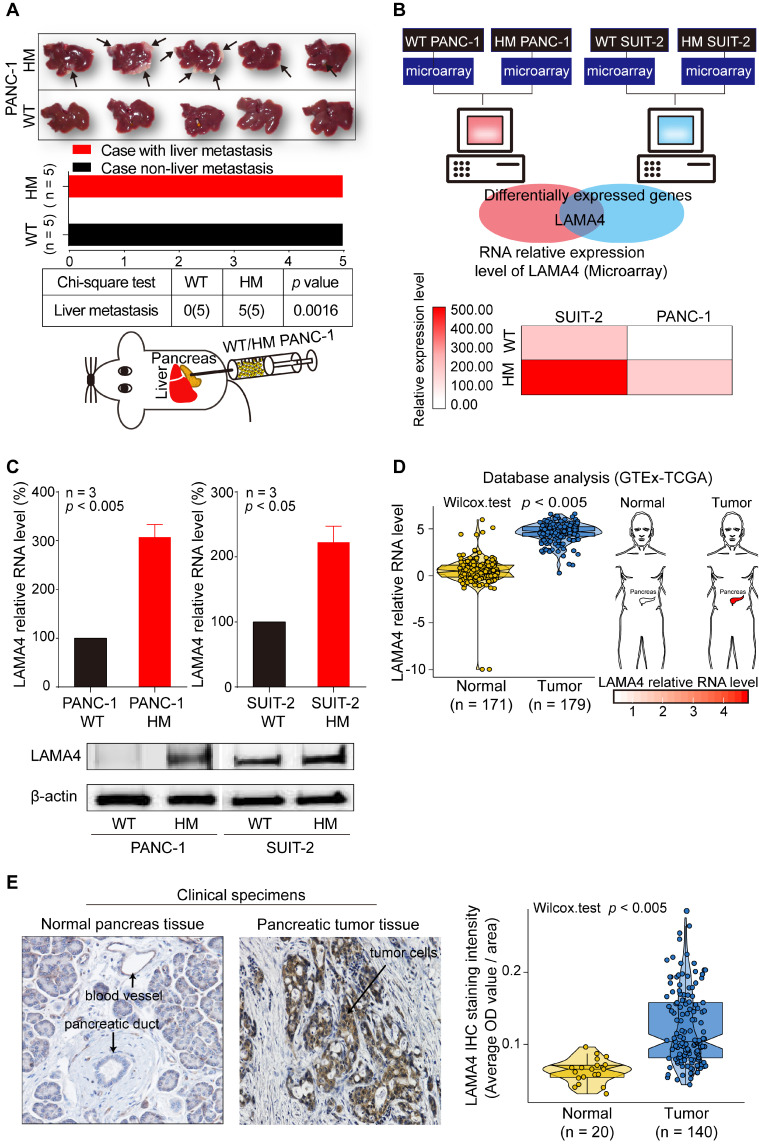
** Establishment of liver highly metastatic pancreatic cancer cell lines and comparison of gene expression patterns between WT and HM pancreatic cancer cell lines.** (**A**) Metastatic tumor formation in liver in orthotopic xenograft mice established by transplanting WT or HM PANC-1 cells into pancreas of nude mice. All mice implanted with pancreatic cancer cell lines developed palpable tumors within 49 days after implantation. The metastatic tumors on liver were recognized as white nodules on the periphery of liver and are indicated by arrows. (**B**) Identification of the gene expression signature for liver metastasis between WT and HM pancreatic cancer cell lines. Gene expression profiles revealed a pancreatic cancer liver metastasis-related gene, the *LAMA4* gene. The *LAMA4* gene was identified as a target gene by taking the intersection from differentially expressed genes in HM PANC-1 and HM SUIT-2 pancreatic cancer cell lines. Microarray analysis showed that *LAMA4* was highly expressed in HM cell lines compared with WT cell lines. (**C**) qRT-PCR analysis of *LAMA4* mRNA expression in WT and HM PANC-1 and SUIT-2 pancreatic cancer cell lines. Gene expression was normalized against *GAPDH* mRNA levels. Western blot analysis of LAMA4 protein expression in WT and HM PANC-1 and SUIT-2 pancreatic cancer cell lines. (**D**) Conjoint analysis of GTEx and TCGA datasets. The datasets of normal samples and pancreatic tumor samples were generated by GTEx and TCGA databases and normalized by UCSC Xena for comparison. *LAMA4* mRNA expression level was extracted and compared. (**E**) IHC staining of LAMA4 in pancreatic tumor tissue and normal pancreas tissue. The quantification of LAMA4 was carried out and compared between normal and tumor tissues.

To investigate the underlying mechanisms of liver metastasis in pancreatic cancer, we established a HM human pancreatic cancer cell line, HM PANC-1, by *in vivo* selection. We then examined the liver-metastatic ability of WT and HM PANC-1 cell lines *in vivo*. WT PANC-1 and HM PANC-1 cell lines were orthotopically transplanted into the pancreas of nude mice. As shown in Figure 1A, HM PANC-1 cells showed much stronger capacity for liver metastasis than WT PANC-1 cells (Chi-square test; *p* = 0.0016). The stronger liver-metastatic ability of HM PANC-1 cells was validated by CT scan (Figure S2A).

To investigate the genes that are involved in liver metastasis of pancreatic cancer, we performed microarray analysis in human pancreatic WT and HM PANC-1 cells as well as human pancreatic WT and HM SUIT-2 cells. The HM SUIT-2 cell line was established and characterized in our previous study 20. We identified a metastasis-related gene, *LAMA4*, that was upregulated in both HM SUIT-2 and HM PANC1 cell lines compared to the corresponding WT cell lines (Figure 1B). To confirm the reliability of microarray data, we next performed quantitative RT-PCR (qRT-PCR) and western blot to examine *LAMA*4 mRNA and protein levels in WT and HM pancreatic cancer cell lines. LAMA4 was highly expressed in HM PANC-1 and HM SUIT-2 at both the mRNA and protein levels (Figure 1C).

To further investigate *LAMA4* gene expression pattern in pancreatic cancer, we next performed TCGA-GTEx conjoint database analysis to compare *LAMA4* RNA expression between normal pancreatic tissues and pancreatic tumor tissues. As shown in Figure 1D, *LAMA4* mRNA expression was significantly increased in pancreatic tumor tissues compared with normal pancreatic tissues (Wilcox test; *p* < 0.005). To validate LAMA4 increase in pancreatic tumor tissues, we next examined LAMA4 protein expression in isolated human clinical specimens of pancreatic tumor tissue and normal tissue using IHC staining. LAMA4 was overexpressed in tumor tissues compared to normal tissues (Figure 1E) in pancreatic cancer.

### LAMA4 correlates with pancreatic cancer liver metastasis, histologic grade and clinical survival of patients

**Figure 2:**
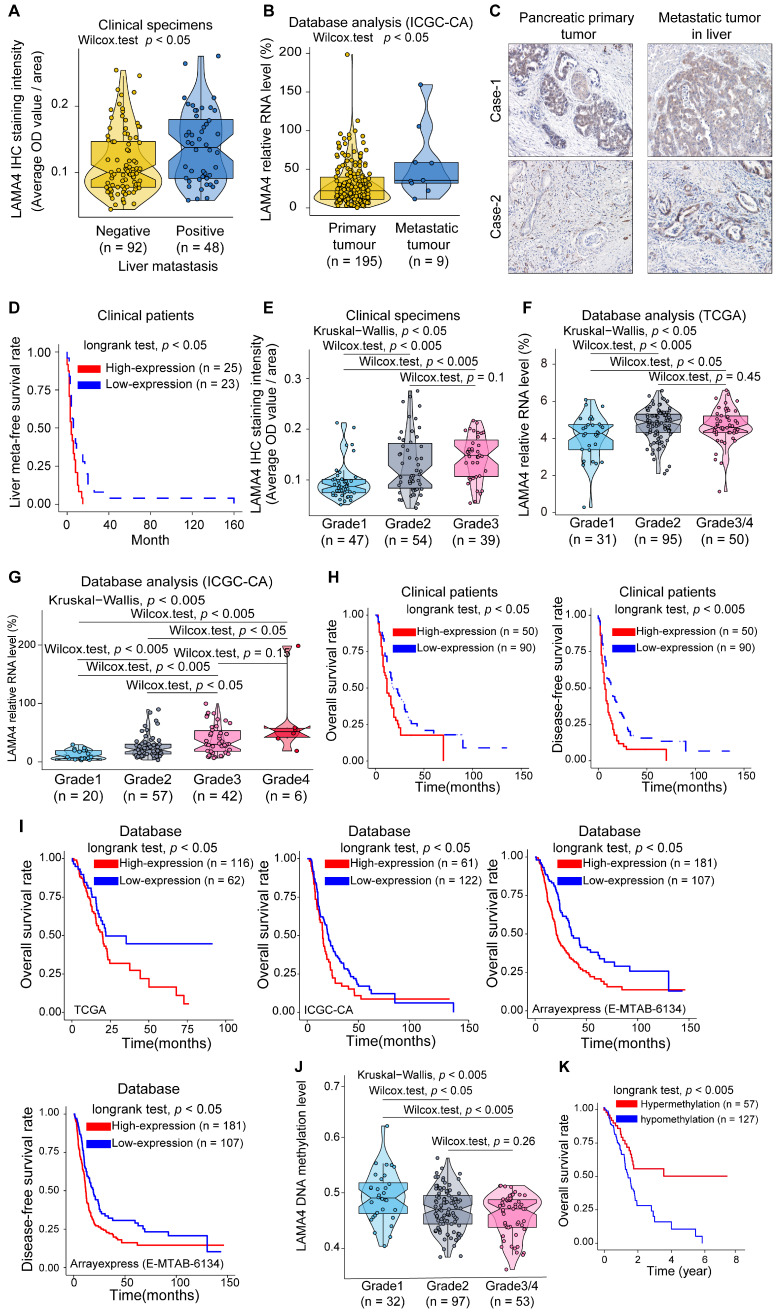
** Association between LAMA4 expression and pancreatic cancer severity.** (**A**) LAMA4 protein expression as determined by IHC in pancreatic cancer patients without or with liver metastasis. (**B**) *LAMA4* mRNA expression between pancreatic tumor patients with and without metastasis. The dataset was downloaded from the ICGC database. (**C**) IHC staining of LAMA4 protein in primary tumor tissues and paired liver metastatic tissues from pancreatic cancer patients. (**D**) Kaplan-Meier curves for liver meta-free survival rate of patients according to LAMA4 levels as determined by IHC. (**E**) Association between LAMA4 protein expression determined by IHC and pancreatic tumor histologic grade. (**F**) Association between *LAMA4* mRNA expression level and pancreatic tumor histologic grade. mRNA expression data were downloaded from TCGA databases. (**G**) Association between *LAMA4* mRNA expression level and pancreatic tumor histologic grade. mRNA expression data were downloaded from ICGC databases. (**H**) Kaplan-Meier curves of 140 pancreatic cancer patients according to LAMA4 expression as detected by IHC. (**I**) Kaplan-Meier curves for pancreatic cancer patients according to *LAMA4* mRNA expression levels downloaded from TCGA, ICGC and ArrayExpress databases. (**J**) Association between *LAMA4* DNA methylation status and pancreatic tumor histologic grade. The TCGA dataset was used for the analysis. (**K**) Kaplan-Meier survival curves for pancreatic cancer patients according to *LAMA4* DNA methylation status. The TCGA dataset was used for the analysis.

To explore the clinical role of LAMA4 in pancreatic cancer, we examined the association between LAMA4 and clinicopathological factors in 140 pancreatic cancer patients. IHC staining was performed to evaluate LAMA4 protein expression in tumor tissues. As shown in Table 1, The LAMA4 high expression group was more likely to have liver metastasis (chi-square; *p* = 0.003). Meanwhile, pancreatic cancer patients with liver metastasis tended to have relatively higher LAMA4 protein expression in the primary tumor compared with patients without liver metastasis (Figure 2A, Wilcox test; *p* = 0.012). To further validate the relationship between LAMA4 and metastasis, we next examined the relationship between LAMA4 and metastasis in other cohorts of pancreatic cancer patients. We compared mRNA level of LAMA4 between primary tumors and non-matched metastatic tumors using the ICGC database. We found that metastatic tumor tissues were associated with higher *LAMA4* expression compared with primary tumor tissues (Figure 2B, Wilcox test; *p* = 0.017). We then examined LAMA4 expression in primary pancreatic tumor tissues and paired liver-metastatic tumor tissues by IHC staining. Liver-metastatic tumor tissues showed higher LAMA4 expression compared to paired primary tumor tissues (Figure 2C). Furthermore, analysis of the liver meta-free survival rate after pancreatectomy showed that pancreatic cancer patients with high *LAMA4* expression showed earlier liver metastasis compared to patients with low *LAMA4* expression (Figure 2D, logrank test; *p* = 0.013). These results support the role of LAMA4 in liver metastasis and suggested that pancreatic cancer patients with high LAMA4 expression were more prone to liver metastasis.

The LAMA4 high expression group was accompanied by higher tumor histologic grade (Table 1, chi-square; *p* < 0.001). Meanwhile, we observed a positive correlation between LAMA4 relative expression and tumor histologic grade in patient samples (Figure 2E). We next used database-based analysis to validate these results. As shown in Figure 2F and Figure 2G, *LAMA4* mRNA expression increased with tumor histologic grade in both TCGA and ICGC datasets, consistent with the LAMA4 IHC results in Figure 2E. The correlation between *LAMA4* and histologic grade was also observed in logistics regression analysis (Figure S2B). The bioinformatic results reinforced the positive correlation between *LAMA4* and histologic grade. These findings suggest that upregulation of *LAMA4* is positively correlated with pancreatic tumor histologic grade.

We then assessed the association between LAMA4 expression and survival of 140 patients. As shown in Figure 2H, both overall survival and disease-free survival determined by Kaplan-Meier analysis revealed that high LAMA4 protein expression was associated with significantly shorter survival (logrank test; overall survival, *p* = 0.026 and disease-free survival, *p* = 0.0031). To validate the survival curves of the clinical patients, we also performed database-based survival analysis for LAMA4. A similar trend of Kaplan-Meier curves was observed. As shown in Figure 2I, significantly worse survival was observed for patients with high *LAMA4* expression in TCGA (logrank test; *p* = 0.045), ICGC (longrank test; *p* = 0.047) and ArrayExpress datasets (logrank test; overall survival rate, *p* = 0.001 and disease-free survival rate,* p* = 0.006). These observations suggest that LAMA4 overexpression is negatively associated with survival in patients.

Furthermore, we examined the relation between *LAMA4* DNA methylation status and pancreatic tumor histologic grade and survival by using TCGA dataset. Notably, *LAMA4* DNA methylation levels were negatively correlated with tumor histologic grade in pancreatic cancer patients (Figure 2J). These results were consistent with our observation above, which suggest that LAMA4 increase, as well as increased tumor histologic grade, is likely regulated by gene methylation. We also performed Kaplan-Meier survival curve analysis according to *LAMA4* DNA methylation status and found that methylation level of *LAMA4* DNA was positively correlated with survival (Figure 2K, logrank test; *p* = 0.005). These results suggest that *LAMA4* DNA methylation status is associated with tumor histologic grade and survival of pancreatic cancer patients. Therefore, modulating *LAMA4* methylation may thus serve as a potential strategy for manipulation of expression of *LAMA4*.

### Decreased expression of LAMA4 inhibits pancreatic cancer liver metastasis *in vivo*

**Figure 3:**
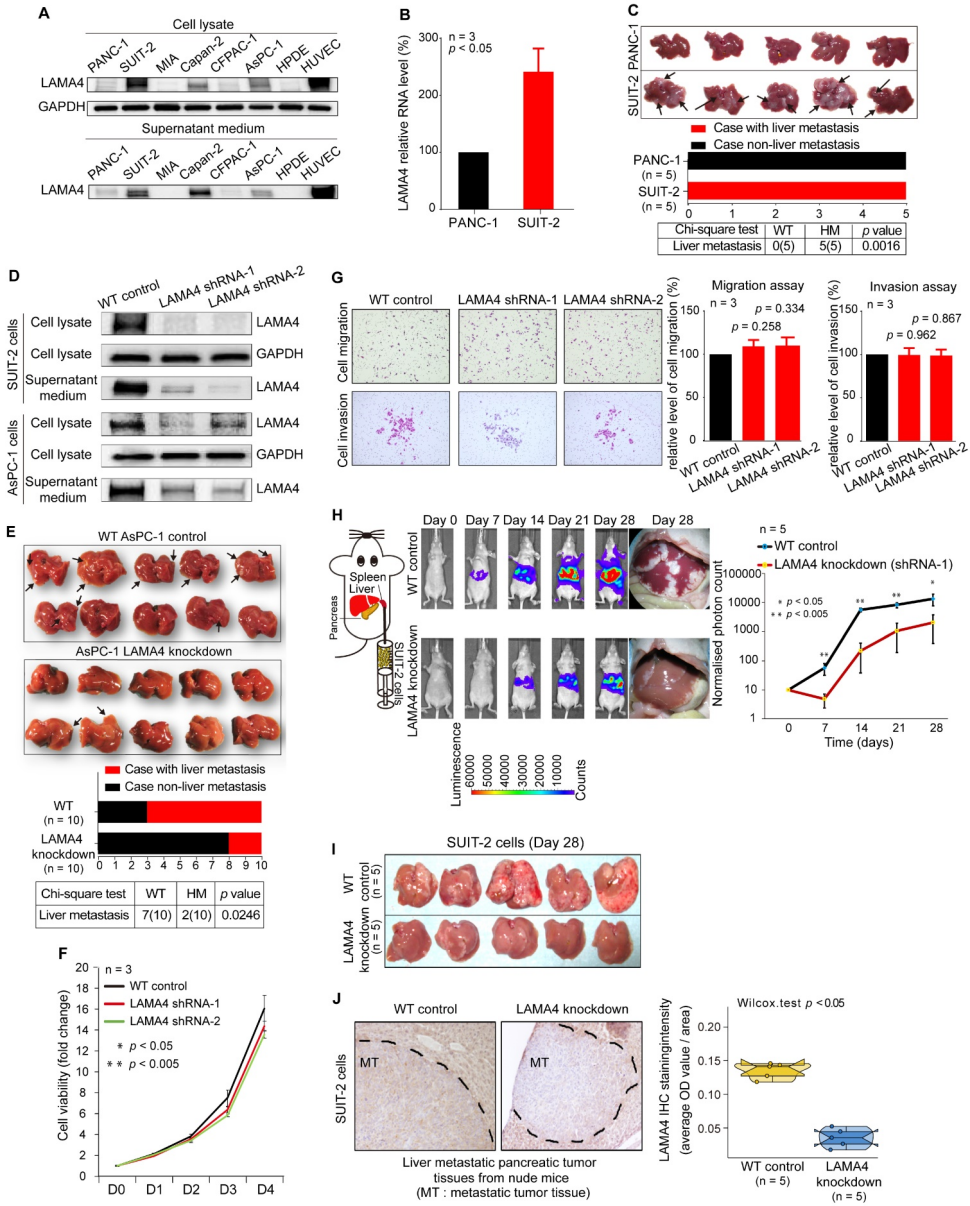
** Association between LAMA4 expression and liver metastasis.** (**A**) Western blot analysis of LAMA4 protein expression in cell lysates and supernatant medium from various human pancreatic cancer cell lines. The HPDE cell line was used as a negative control for LAMA4 expression, and the HUVEC line was used as a positive control. (**B**) qRT-PCR analysis of *LAMA4* mRNA levels in PANC-1 and SUIT-2 cells. Gene expressions were normalized against *GAPDH* mRNA levels. Error bars, SEM (n=3). (**C**) Metastatic tumor colonization on liver in orthotopic xenograft mice established by transplanting SUIT-2 and PANC-1 cells into pancreas of nude mice. All mice implanted with pancreatic tumor cell lines developed palpable tumors after implantation. Metastatic tumors on liver were recognized as white nodules on the periphery of liver and are indicated by arrows. (**D**) Western blot analysis of LAMA4 protein expression in cell lysates and supernatant medium in WT and LAMA4-depleted pancreatic cancer cell lines. (**E**) Metastatic tumor colonization on liver in orthotopic xenograft mice established by transplanting WT AsPC-1 or LAMA4-depleted AsPC-1 cells into pancreas of nude mice. All mice implanted with pancreatic cancer cells developed palpable tumors after implantation. Metastatic tumors on liver are indicated by arrows. (**F**) The effects of LAMA4 knockdown on cell viability was measured in SUIT-2 cells. (**G**) The effects of LAMA4 knockdown on cell migration and invasion were examined in SUIT-2 cells. LAMA4 knockdown did not affect cell migration and invasion in vitro. (**H**) *In vivo* IVIS images of tumor growth of luciferase-expressing SUIT-2 cells (WT or LAMA4-depleted) implanted in spleen of mice after the indicated times. Tumor tissues on liver were recognized as white nodules on liver. Quantitative comparison of signals from the IVIS luciferase images was performed. (**I**) Liver tumor colonization condition on day 28 of IVIS examination. The tumors on liver were recognized as white nodules on liver (**J**) IHC staining confirming successful downregulation of LAMA4 in tumor tissues in livers. Quantification of LAMA4 staining was carried out for comparison.

To investigate the effect of LAMA4 in liver metastasis of pancreatic cancer cells, we first examined LAMA4 protein expression in pancreatic cancer cell lines. LAMA4 is highly expressed in SUIT-2 cells and expressed at lower levels in PANC-1 cells at both the protein and RNA levels (Figure 3A and B). Secreted LAMA4 was detected in culture medium by western blot (Figure 3A). We next examined the liver metastatic ability of SUIT-2 cells and PANC-1 cells *in vivo*. The two cell lines were orthotopically transplanted into the pancreas of nude mice. As shown in Figure 3C, SUIT-2 cells showed a significantly stronger capacity for liver metastasis, as compared to PANC-1 cells (chi-square; *p* = 0.0016). These results suggested that SUIT-2 cells in which LAMA4 was highly expressed were more prone to liver metastasis.

We next stably downregulated LAMA4 expression by shRNA in SUIT-2 and AsPC-1 human pancreatic cancer cells and confirmed downregulated LAMA4 at both mRNA (Figure S3A) and protein (Figure 3D) levels. We then examined the effects on LAMA4 knockdown on the liver metastasis ability of pancreatic cancer cells *in vivo*. We injected WT or LAMA4-depleted AsPC-1 cells into the pancreas of nude mice. Notably, LAMA4 depletion in AsPC-1 cells significantly reduced the capacity of liver metastasis compared with WT AsPC-1 cells (Figure 3E, chi-square; *p* = 0.0246). These results suggest that downregulation of LAMA4 could inhibit the liver-metastatic potential of pancreatic cancer cells *in vivo*. The initial step of tumor metastasis is accompanied by the changes of cancer cell viability, migration and invasion. To further investigate the role of LAMA4 in metastasis, we proceeded to explore the potential effects of LAMA4 in cell viability, migration and invasion. LAMA4 downregulation did not cause any effect on pancreatic cancer cell viability, migration or invasion in SUIT-2 cells (Figure 3F and G) and AsPC-1 cells (Figure S3B and C).

Furthermore, we examined the effect of LAMA4 on liver colonization. The luciferase-expressing SUIT-2 or AsPC-1 cells (WT or *LAMA4-*depleted) were directly injected into the spleen of nude mice, and IVIS *in vivo* imaging was used for luminescence visualization of fluorescence-labeled tumors at various days after implantation. As shown in Figure 3H and Figure S3D, both *LAMA4*-depleted SUIT-2 and AsPC-1 cells showed decreased capability of tumor formation in liver compared with WT SUIT-2 and AsPC-1 cells. The pancreatic tumors on liver was smaller in the *LAMA4*-depleted SUIT2 and AsPC-1 group than in the WT control group on day 28 of IVIS examination (Figure 3I and Figure S3E). *LAMA4* knockdown in metastatic tumor tissues on liver was validated by IHC staining (Figure 3J and Figure S3F). These findings suggest that LAMA4 is highly correlated with liver metastasis phenotype and that downregulation of LAMA4 inhibits pancreatic cancer liver colonization *in vivo*.

### TME alterations are associated with pancreatic cancer liver metastasis

**Figure 4:**
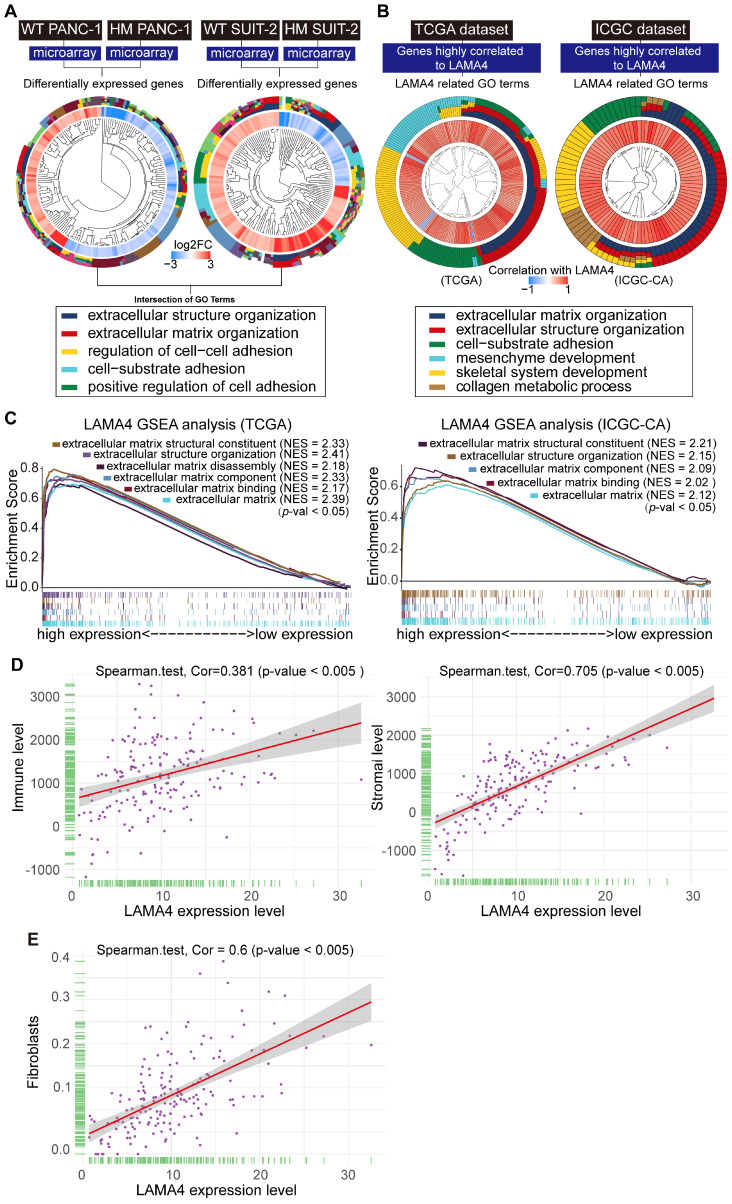
** Functional annotation of gene expression signatures between WT and HM pancreatic cancer cell lines.** (**A**) GO enrichment analysis of differentially expressed genes between WT and HM pancreatic cancer cell lines. Gene expression profiling data of WT and HM pancreatic cancer cell lines were analyzed and differentially expressed genes were identified (|logFC |> 1, p < 0.05). The packages used for R program were “clusterProfiler,” “org.Hs.eg.db,” “enrichplot” and “ggplot2.” The setup of parameters for r script were pvalueCutoff =0.05 and qvalueCutoff = 0.05. (**B**) *LAMA4*-related gene ontology terms. Genes that strongly correlated with *LAMA4* were screened by Spearman's correlation analysis (Spearman |R| > 0.4, p < 0.05) based on the TCGA and ICGC datasets. We then explored the biofunction of the *LAMA4*-related genes by GO analysis. (**C**) The median of LAMA4 RNA expression was used as a threshold. Patients were assigned into LAMA4 high and LAMA4 low expression groups according to the threshold. Enrichment plots of gene expression signatures of TME-related pathways were selected according to the difference between LAMA4 high and LAMA4 low expression groups. The dataset was downloaded from the TCGA and ICGC databases. (**D**) Correlation analysis between stromal cell infiltration and LAMA4 RNA expression as well as immune cell infiltration and *LAMA4* RNA expression. Stromal cell infiltration and immune cell infiltration were evaluated by an R package ESTIMATE. TCGA dataset was download for the analysis. Spearman's correlation test was performed to examine the correlation coefficient. (**E**) Correlation analysis between *LAMA4* RNA expression and CAFs level. CAFs infiltration level was evaluated using xCell database. Spearman's correlation test was performed to examine the correlation coefficient.

To clarify the functions of gene expression signatures associated with pancreatic cancer liver metastasis, we performed gene ontology (GO) analysis of differentially expressed genes between WT and HM PANC-1 cell lines as well as those between WT and HM SUIT-2 cell lines. The R package clusterprofiler was used for the analysis 26. As shown in Figure 4A and Figure S4A, the intersection of GO terms showed that a proportion of differentially expressed genes was associated with extracellular environment alterations which is a major structural component of the TME. These results suggested that TME alterations might contribute to pancreatic cancer liver metastasis.

We next explored the biologic role of LAMA4 in pancreatic cancer. We observed that genes that highly correlated with *LAMA4* were involved in TME alterations in both TCGA and ICGC databases (Figure 4B and Figure S4B). To further explore the role of *LAMA4*, we conducted (GSEA) of low and high *LAMA4* expression datasets. GSEA analysis also showed significant differences (*p* < 0.05) in enrichment of TME changes in *LAMA4* high expression phenotype in both TCGA and ICGC databases (Figure 4C). Given the importance of TME in liver metastasis and the impact of LAMA4 on TME, we suggest that liver metastasis in pancreatic cancer may be influenced by LAMA4-mediated TME alterations.

We next sought to examine the relationship between *LAMA4* and the pancreatic TME. Immune cell and stromal cell infiltration levels were characterized using an R package ESTIMATE 27. The correlations between Immune cell/stromal cell infiltration levels and *LAMA4* RNA level were examined by correlation test (Figure 4D). Notably, we observed a positive correlation (r = 0.705) between *LAMA4* and stromal cell infiltration. As CAFs are a major component of the pancreatic cancer stroma and contribute to metastasis in pancreatic cancers 28, we next calculated the correlation between *LAMA4* expression and infiltration level of CAFs. The infiltration level of CAFs was evaluated by using xCell database 29. As shown in Figure 4E, *LAMA4* expression showed a positive correlation with CAFs (r = 0.6). These data indicate that LAMA4 may play a role in mediating the TME response and might be involved in CAFs infiltration in pancreatic tumors.

### Decreased expression of LAMA4 in pancreatic tumor cells reduces recruitment and activation of CAFs

**Figure 5:**
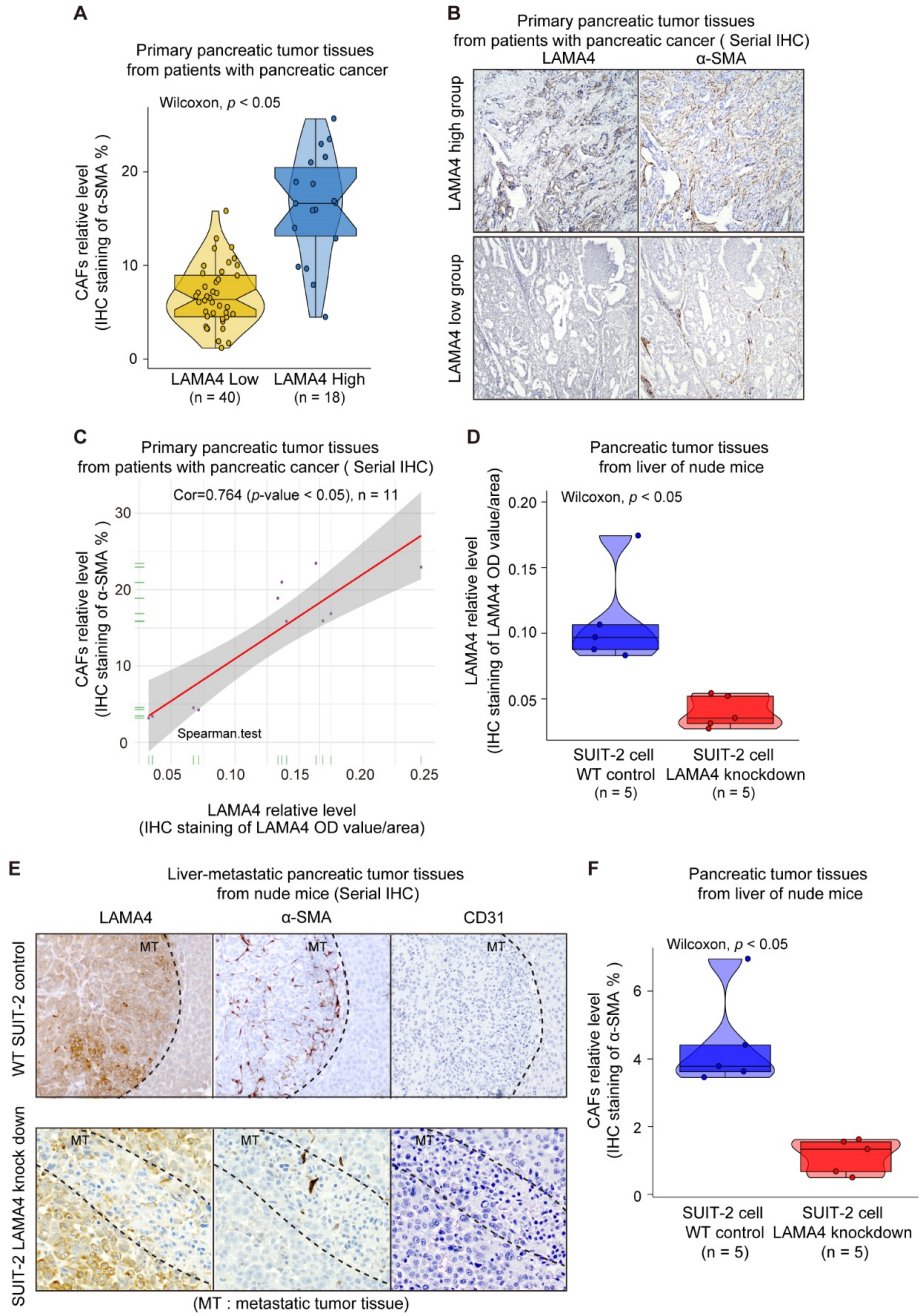
** Validation of correlation between LAMA4 expression and CAFs recruitment *in vivo*.** (**A**) Comparison of CAFs level as determined by IHC staining in primary pancreatic tumor tissues from patients with pancreatic cancer. The CAFs in the LAMA4 high expression group (n=18) were higher than that in the LAMA4 low expression group (n=40). (**B**) Serial IHC staining for LAMA4 and CAFs in primary pancreatic tumor tissues from patients with pancreatic cancer. The CAFs level in LAMA4 high expression group was higher than that in the LAMA4 low expression group. (**C**) Correlation analysis between CAFs and LAMA4 protein level as determined by serial IHC staining in patient samples. LAMA4 protein expression level and CAFs level were semi-quantitatively scored according to IHC staining intensity. Spearman's correlation test was performed to examine the correlation coefficient. (**D**) Quantitative values of IHC staining confirmed LAMA4 knockdown in WT SUIT-2-induced tumor tissues or LAMA4-depleted SUIT-2-induced tumor tissues on liver of nude mice. (**E**) Serial IHC staining for LAMA4, CAFs and blood vessel (CD31) in metastatic tumor tissues from liver of nude mice. (**F**) IHC quantitative values of CAFs in metastatic tumor tissues from liver of nude mice. The CAFs in the LAMA4 knockdown group were lower than that in the LAMA4 WT group.

**Figure 6:**
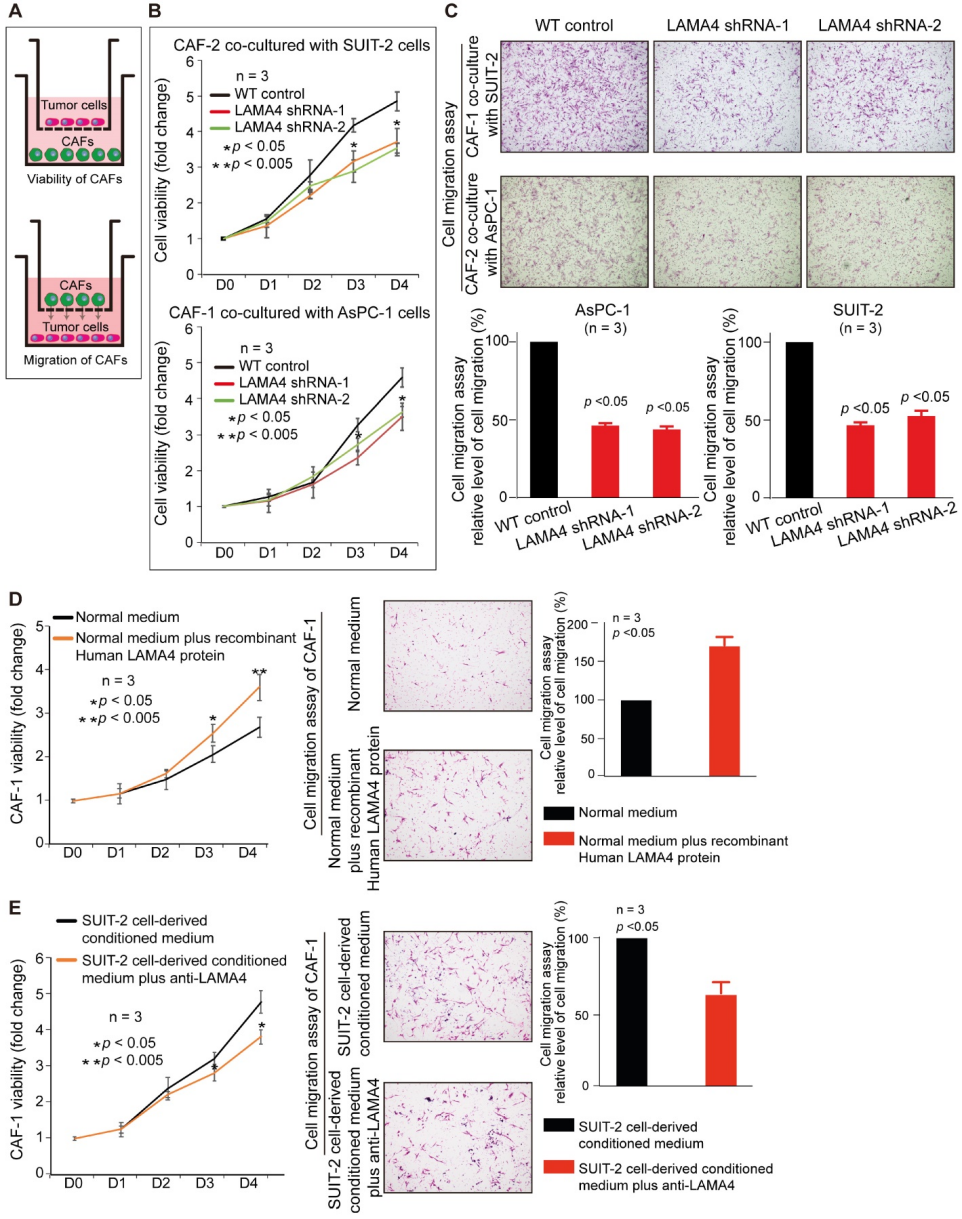
** Validation of relationship between cancer cell-derived secreted LAMA4 and CAFs recruitment/activity *in vitro*.** (**A**) Schematic representation of non-contact co-culture. (**B**) Cell viability assay of CAFs was performed after non-contact co-culture with either WT pancreatic cancer cells or pancreatic cancer cells subjected to LAMA4 knockdown. (**C**) Cell migration assay of CAFs was performed after non-contact co-culture with either WT pancreatic cancer cells or pancreatic cancer cells subjected to LAMA4 knockdown. (**D**) Cell viability assay and migration assay of CAFs which cultured in normal medium or treated with recombinant human LAMA4 protein. (**E**) Cell viability assay and migration assay of CAFs which incubated in SUIT-2 cells-derived conditioned medium or SUIT-2 cells-derived conditioned medium with anti-LAMA4 antibody.

To investigate the relationship between LAMA4 and CAFs, IHC staining was used to evaluate the level of CAFs in 58 patient samples that were randomly selected from the 140 human primary pancreatic tumor tissues. We found that LAMA4 high expression tumor tissues showed a significantly higher level of CAFs compared to LAMA4 low expression patient samples (Figure 5A, Wilcoxon test; *p* < 0.005). To further validate the positive correlation between LAMA4 and CAFs, the distributions of LAMA4 protein and CAFs (α-SMA) were examined by serial IHC staining in human primary pancreatic tumor tissues. As shown in Figure 5B, LAMA4 high expression tumor tissue was accompanied by a high level of CAFs, whereas LAMA4 low expression patient samples showed a significantly low level of CAFs. We then calculated the correlation coefficient between LAMA4 protein level and concomitant CAFs level determined by serial IHC and observed a highly positive correlation between LAMA4 and CAFs (Figure 5C, Cor = 0.764). We also assessed the relationship between LAMA4 and CAFs in nude mice. All mice implanted with WT or *LAMA4*-depleted SUIT-2 cells in pancreas developed palpable tumors after implantation. LAMA4 protein expression and CAFs (α-SMA) were semi-quantitatively scored according to IHC staining intensity in liver-metastatic tumor tissues. LAMA4 knockdown in metastatic tumor tissues on liver was confirmed (Figure 5D). Distributions of LAMA4 protein and CAFs were examined by serial IHC staining in liver metastatic tumor tissues in nude mice. As shown in Figure 5E, LAMA4 protein expression and distribution highly coincide with CAFs on mice liver-metastatic tumor tissues determined by serial IHC staining (α-SMA positive areas did not express the blood vessel marker CD31). Notably, LAMA4 knockdown was accompanied with a significant reduction of CAFs (Figure 5F, *p* = 0.0079). To further determine the influence of secreted LAMA4 knockdown in pancreatic cancer cell on CAFs viability and migration, a non-contact co-culture was performed. The CAFs and WT or *LAMA4*-depleted pancreatic cancer cell lines were co-cultured respectively. The effects of LAMA4 knockdown in pancreatic cancer cells on CAFs viability and migration were evaluated (Figure 6A, B and C). As shown in Figure 6B, CAFs exhibited a significantly decreased viability where co-cultured with *LAMA4*-depleted SUIT-2 cells or AsPC-1 cells. Meanwhile, lower migration ability of CAFs was observed where co-cultured with *LAMA4*-depleted SUIT-2 cells or AsPC-1 cells (Figure 6C). To further confirm the effect of secreted LAMA4 on CAFs, we treated CAFs with purified recombinant human LAMA4 protein. Our results demonstrated that the purified recombinant LAMA4 protein increased the viability and migration ability of CAFs (Figure 6D). Next, we specifically downregulated LAMA4 protein in conditioned medium from SUIT-2 cells using anti-LAMA4 antibody. We observed that LAMA4 downregulation in conditioned medium inhibited the viability and migration ability of CAFs (Figure 6E). These results suggest that LAMA4 secreted from pancreatic cancer cells has a positive influence on the viability and migration of CAFs.

## Discussion

Desmoplasia is one of the hallmarks of pancreatic cancer 30. The aberrant proliferation of stromal cells, such as fibroblasts, and increased deposition of altered matrix proteins create an environment that facilitates pancreatic cancer growth, metastasis and drug resistance. In particular, multiple matrix proteins, such as collagen and fibronectin, are highly produced by pancreatic cancer cells 31. Matrix proteins that are abnormally expressed in pancreatic cancer are associated with pancreatic cancer progression. In our study, pancreatic cancer cells overexpressed LAMA4, which is a component of endothelial basement membranes, and its expression highly correlated with pancreatic tumor histologic grade and survival of patients.

The fast progression and high metastasis ability of pancreatic cancer results in poor prognosis and increases the death rate of patients 1. In pancreatic cancer diagnosis, the major concern is whether the tumor has metastasized, which determines if the cancer can be surgically removed. To date, there are no specific biomarkers for pancreatic cancer progression 32. Therefore, identification of specific molecules that indicate early cancer stages or mediate liver metastasis could facilitate and improve the development of current clinical therapies for pancreatic cancer 33. In our study, both *LAMA4* RNA and LAMA4 protein levels were highly correlated with pancreatic tumor histologic grade and patient survival in both clinical tumor tissues and bioinformatic analysis. The correlation between LAMA4 and tumor severity might contribute to poor prognosis in patients with pancreatic cancer. We found that *LAMA4* DNA hypomethylation was associated with poor disease prognosis. Notably, a previous study showed that miRNA-200b regulates metastasis via targeting LAMA4 in renal cell carcinoma 34. Based on these findings, we suggest that understanding the epigenetic modifications of LAMA4 could be of clinical relevance.

A previous study showed that LAMA4 promotes tumor re-initiation and metastatic cell proliferation and colonization in breast cancer 19. In the current study, we demonstrate for the first time that LAMA4 is involved in pancreatic cancer metastasis. Notably, a positive correlation was observed between LAMA4 expression and CAFs in both clinical patient tissues and bioinformatic analyses. We also demonstrated that LAMA4 downregulation resulted in a reduction of CAFs in metastatic tumors in nude mice. Our non-contact co-culture results indicated that LAMA4 knockdown in pancreatic cancer cells has a negative influence on the viability and migration of CAFs. Furthermore, the purified recombinant LAMA4 protein showed positive effects on promoting the viability and migration ability of CAFs. Specific downregulation of cancer cell-derived secreted LAMA4 inhibited the viability and migration of CAFs. These findings suggest that pancreatic cancer cell-derived secreted LAMA4 has positive effects on CAFs. Previous studies have shown that the extracellular microenvironment modulates fibroblast growth 35. Based on the association between *LAMA4* and the extracellular microenvironment in our bioinformatic analysis, we proposed that CAFs infiltration in pancreatic cancer might be influenced by LAMA4-mediated extracellular microenvironment alteration. CAFs are a major component of the pancreatic cancer stroma 28 and contribute to cancer cell invasion, angiogenesis and metastasis in various cancers 36-38. Previous studies showed that CAFs promote pancreatic tumor progression 6, 7, 36, accelerate invasion and metastasis of pancreatic tumors, and promote proliferation and growth of pancreatic cancer cells 8. In particular, during pancreatic cancer liver metastasis, CAFs result in a fibrotic microenvironment on liver that promotes metastatic tumor growth 39-41. We propose the possibility that pancreatic cancer cell-derived secreted LAMA4 could influence the recruitment or activation of CAFs, resulting in a favorable microenvironment that sustains pancreatic cancer metastasis or metastatic tumor growth in liver. We suggest that LAMA4-mediated CAFs recruitment and activation may contribute to pancreatic cancer metastasis. Interference with the recruitment or activation of CAFs might be achieved via LAMA4 manipulation, which might provide a potential therapeutic approach to pancreatic cancer.

LAMA4 overexpression in aggressive pancreatic cancer and its metastatic potential as well as the relationship of LAMA4 to CAFs provide good prospects to our study. Not only does our work help provide insights into the mechanism(s) of LAMA4 upregulation and its association with advanced pancreatic cancer, it also holds the promise of yielding potential biomarker(s) and therapeutic targets for an improved management of pancreatic cancer. Nevertheless, the molecular mechanism of the LAMA4-CAFs interaction is still unclear and thus warrants further research to determine the potential mechanisms of LAMA4-mediated CAFs recruitment and activation.

## Supplementary Material

Supplementary figures and tables.

## Figures and Tables

**Table 1:** Relationship between LAMA4 expression and various clinicopathological factors in patients with pancreatic ductal adenocarcinoma (n = 140)

Characteristics	Low expression group, N =90 (64.3%)	High expression group, N =50 (36.7%)	*P* value
**Age**			0.450
< 65	42 (46.7)	20 (40.0)	
≥ 65	48 (53.3)	30 (60.0)	
**Sex**			0.898
Female	35 (38.9)	20 (40.0)	
Male	55 (61.1)	30 (60.0)	
**pT category**			0.688
pT1 / pT2	4 (4.4)	3 (6.0)	
pT3 / pT4	86 (95.6)	47 (94.0)	
**pN category**			0.188
pNo	21 (23.31)	7 (14.0)	
pN1	69 (76.7)	43 (86.0)	
**UICC staging**			0.523
I	3 (3.3)	1 (2.0)	
II	79 (87.8)	47 (94.0)	
III / IV	8 (8.9)	2 (4.0)	
**Residual tumor**			0.507
R0	61 (67.8)	32 (64.0)	
R1	29 (32.2)	18 (36.0)	
**Histologic grade**			<0.001
Grade 1	44 (48.9)	3 (6.0)	
Grade 2	32 (35.6)	22 (44.0)	
Grade 3	14 (15.6)	25 (50.0)	
**Lymphatic invasion (ly)**			0.706
Negative	23 (25.6)	9 (18.0)	
Positive	67 (74.4)	41 (82.0)	
**Vascular invasion (v)**			0.051
Negative	36 (40.0)	12 (24.0)	
Positive	54 (60.0)	38 (76.0)	
**Perineural invasion (ne)**			0.210
Negative	16 (17.80)	5 (10.0)	
Positive	74 (82.20)	45 (90.0)	
**Liver metastasis**			0.003
Negative	67 (74.4)	25 (50.0)	
Positive	23 (25.6)	25 (50.0)	

## Abbreviations

LAMA4: laminin subunit alpha 4
CAFs: cancer-associated fibroblasts
TME: tumor microenvironment
WT: wild-type
HM: highly liver metastatic
IHC: immunohistochemistry
OD: optical density
HPDE: human pancreatic ductal epithelial
HUVECs: Human umbilical vein endothelial cells
α-SMA: alpha-smooth muscle actin
CD90: cluster of differentiation 90
GFAP: glial fibrillary acidic protein
CK19: cytokeratin 19
GAPDH: glyceraldehyde-3-phosphate dehydrogenase
GO: gene ontology
GSEA: gene set enrichment analysis
ICGC: International Cancer Genome Consortium
GTEx: The Genotype-Tissue Expression
TCGA: The Cancer Genome Atlas Program
