# LAMA4 upregulation is associated with high liver metastasis potential and poor survival outcome of Pancreatic Cancer: Erratum

**DOI:** 10.7150/thno.68023

**Published:** 2021-11-10

**Authors:** Biao Zheng, Jianhua Qu, Kenoki Ohuchida, Haimin Feng, Stephen Jun Fei Chong, Zilong Yan, Yicui Piao, Peng Liu, Nan Sheng, Daiki Eguchi, Takao Ohtsuka, Kazuhiro Mizumoto, Zhong Liu, Shazib Pervaiz, Peng Gong, Masafumi Nakamura

**Affiliations:** 1 Department of General Surgery, Shenzhen University General Hospital / Shenzhen University Clinical Medical Academy, Shenzhen, Guangdong 518055, China.; 2 Department of Surgery and Oncology, Graduate School of Medical Sciences, Kyushu University, Fukuoka 812-8582, Japan.; 3 Department of Physiology, Yong Loo Lin School of Medicine, National University of Singapore (NUS), Singapore 117593.; 4 Advanced Medical Initiatives, Graduate School of Medical Sciences, Kyushu University, Fukuoka 812-8582, Japan.; 5 Hepato-pancreato-biliary Surgery Department, Peking University Shenzhen Hospital, Shenzhen, Guangdong 518055, China.; 6 Department of Critical Care Medicine, National Cancer Center/Cancer Hospital & Shenzhen Hospital, Chinese Academy of Medical Sciences and Peking Union Medical College, Shenzhen, Guangdong 518116, China.; 7 Cancer Center of Kyushu University Hospital, Fukuoka 812-8582, Japan.; 8 NUS Graduate School for Integrative Sciences and Engineering, National University of Singapore, Singapore 117593.; 9 National University Cancer Institute, National University Health System, Singapore 119074.; 10 Carson International Cancer Research Centre, Shenzhen University School of Medicine, Shenzhen, Guangdong 518055, China.

In the original Figure 6C of our article 1, we mistakenly repeatedly used the LAMA4 shRNA-1 knockdown figure (Figure 6C, bottom middle) as the LAMA4 shRNA-2 knockdown figure (Figure 6C, bottom right) in the cell migration assay of CAFs. The Figure 6C should be corrected as follows. The figure corrected in this erratum does not influence any original conclusions in our study. We apologize for any inconvenience or misunderstanding that this error may have caused.

## Figures and Tables

**Figure 6:**
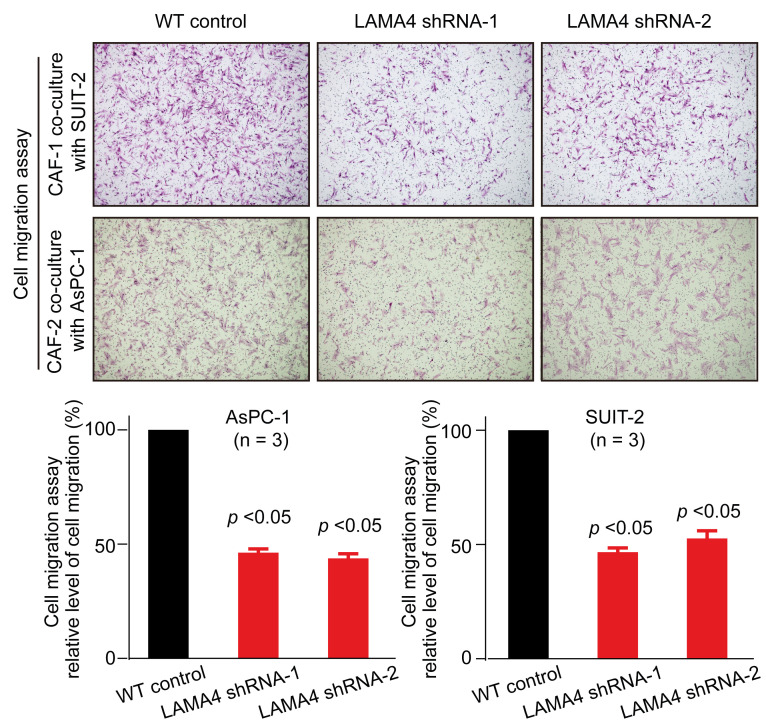
** C**. Cell migration assay of CAFs was performed after non-contact co-culture with either WT pancreatic cancer cells or pancreatic cancer cells subjected to LAMA4 knockdown.

## References

[1] Zheng B, Qu J, Ohuchida K, Feng H, Chong SJF, Yan Z, Piao Y, Liu P, Sheng N, Eguchi D, Ohtsuka T, Mizumoto K, Liu Z, Pervaiz S, Gong P, Nakamura M (2020). LAMA4 upregulation is associated with high liver metastasis potential and poor survival outcome of Pancreatic Cancer. *Theranostics*.

