# Supplementary material for: LAMA4 upregulation is associated with high liver metastasis potential and poor survival outcome of Pancreatic Cancer
Source: Theranostics. 2020 Aug 13;10(22):10274–89. doi: 10.7150/thno.47001 (PMC7481422; doi:10.7150/thno.47001)
Supplement: Supplementary file 1 — Supplementary figures and tables. [file thnov10p10274s1.pdf]

Table S1: Clinicopathological characteristics of patients (n=140)

Median age	66 years (range 36–86 years)
Sex (Male/Female)	85(60.7%) / 55 (39.3%)
pT category	
T1	4(2.9%)
T2	3(2.1%)
T3	129(92.1%)
T4	4(2.9%)
pN category	
pN0	28 (20.0%)
pN1	112 (80.0%)
UICC stage	
I	4(2.8%)
II	126(90.0%)
III	3(2.1%)
IV	7(5.0%)
Residual tumor category	
R0	93 (66.4%)
R1	47 (33.6%)
Histologic grade	
Grade 1	47(33.6%)
Grade 2	54(38.5%)
Grade 3	39(27.9%)
Vascular invasion	
Negative	48 (34.3 %)
Positive	92 (65.7 %)
Perineural invasion	
Negative	21 (15.0%)
Positive	119 (85.0 %)
Lymphatic invasion	
Negative	32 (22.9%)
Positive	108 (77.1%)
Liver metastasis	
Negative	92 (65.7%)
Positive	48 (34.3%)

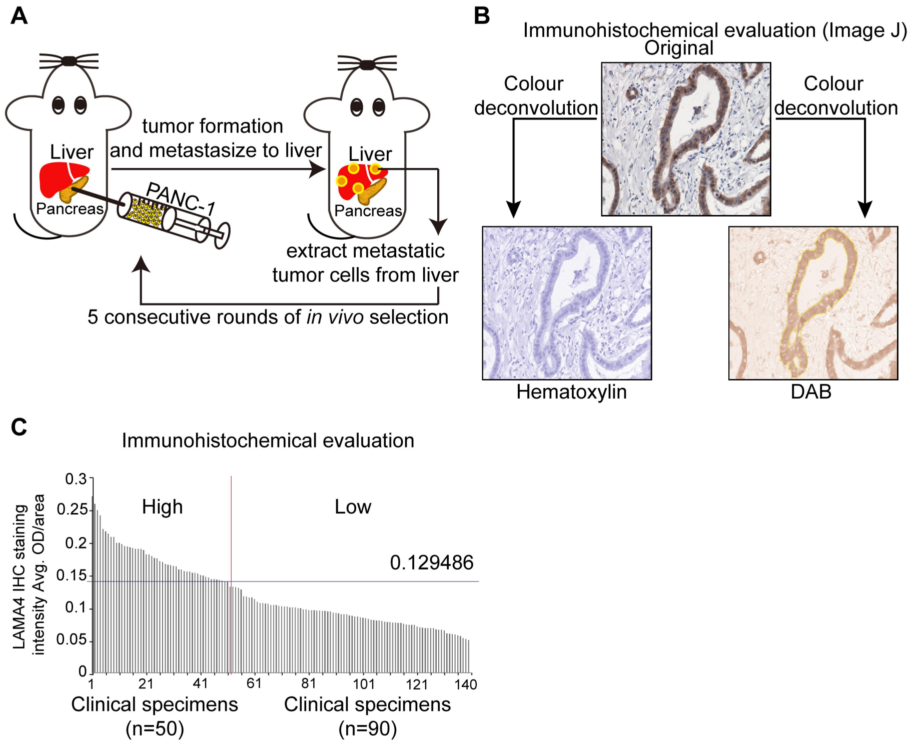

A
Liver
Pancreas
PANC-1
tumor formation
and metastasize to liver
Liver
Pancreas
extract metastatic
tumor cells from liver
5 consecutive rounds of *in vivo* selection
B
Immunohistochemical evaluation (Image J)
Original
Colour
deconvolution
Colour
deconvolution
Hematoxylin
DAB
C
Immunohistochemical evaluation
LAMA4 IHC staining
intensity Avg. OD/area
High
Low
0.129486
0.3
0.25
0.2
0.15
0.1
0.05
0
1
21
41
61
81
101
121
140
Clinical specimens
(n=50)
Clinical specimens
(n=90)

Figure S1. Semi-quantification of LAMA4 expression according to IHC staining intensity. (A) Flow chart of the *in vivo* selection process for HM human pancreatic cancer cell lines. Five consecutive rounds were performed for *in vivo* selection of liver metastasis; metastatic cells were harvested to establish HM PANC-1 cells. (B) DAB and hematoxylin staining results were digitally separated using an ImageJ plugin for color deconvolution. (C) A total of 140 pancreatic cancer tissues were analyzed by LAMA4 IHC staining and Image J. Each spike represents the OD value of an individual pancreatic cancer sample. The average OD value (0.129486) of LAMA4 expressed on blood vessels was used as a threshold. Then, patients were assigned into LAMA4 high and LAMA4 low expression groups according to the threshold. Among the 140 samples, 90 (64.3%) cases were in the high LAMA4 expression group and 50 (36.7%) were in the low LAMA4 expression group.

A

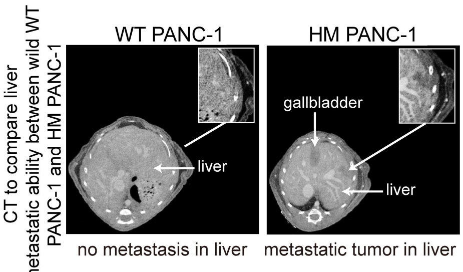

B

Logistic regression analysis

	Tumor histologic grade	Total (N)	Odds ratio in LAMA4 expression	*p*-Value
Clinical	Grade 1 vs Grade 2	101	5.69	0.000166
	Grade 1 vs Grade 3	86	14.07	6.34e-07
TCGA	Grade 1 vs Grade 2	125	3.77	0.00311
	Grade 1 vs Grade 3 / 4	81	1.77	0.24245
ICGC-CA	Grade 1 vs Grade 2	77	7.55	0.01064
	Grade 1 vs Grade 3	62	20.08	0.00024
	Grade 1 vs Grade 4	26	45.00	0.00406

Figure S2. Validation of HM PANC-1 and association between LAMA4 expression and pancreatic cancer histologic grade. (A) Evaluation of metastatic tumor formation in liver by CT scan. (B) Logistic regression analysis testing relationship between LAMA4 expression and tumor histologic grade.

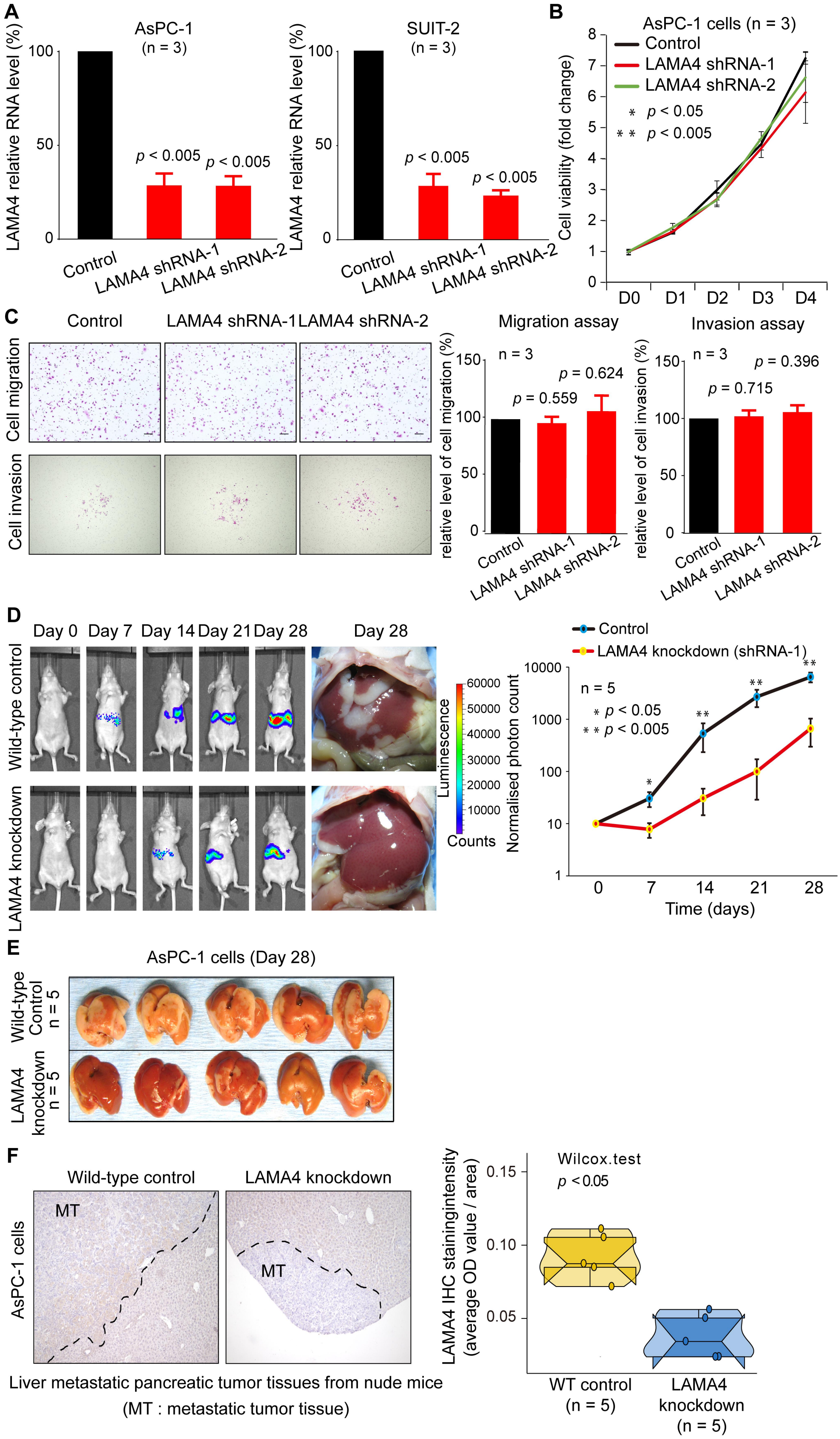
A
AsPC-1
(n = 3)
SUIT-2
(n = 3)
LAMA4 relative RNA level (%)
p < 0.005
Control
LAMA4 shRNA-1
LAMA4 shRNA-2
B
AsPC-1 cells (n = 3)
Control
LAMA4 shRNA-1
LAMA4 shRNA-2
* p < 0.05
** p < 0.005
Cell viability (fold change)
D0
D1
D2
D3
D4
C
Control
LAMA4 shRNA-1
LAMA4 shRNA-2
Cell migration
Cell invasion
Migration assay
Invasion assay
n = 3
p = 0.624
p = 0.559
p = 0.396
p = 0.715
relative level of cell migration (%)
relative level of cell invasion (%)
D
Day 0
Day 7
Day 14
Day 21
Day 28
Wild-type control
LAMA4 knockdown
Luminescence
Counts
Control
LAMA4 knockdown (shRNA-1)
n = 5
* p < 0.05
** p < 0.005
Normalised photon count
Time (days)
E
AsPC-1 cells (Day 28)
Wild-type Control n = 5
LAMA4 knockdown n = 5
F
Wild-type control
LAMA4 knockdown
MT
AsPC-1 cells
Liver metastatic pancreatic tumor tissues from nude mice
(MT : metastatic tumor tissue)
Wilcox.test
p < 0.05
LAMA4 IHC stainingintensity (average OD value / area)
WT control (n = 5)
LAMA4 knockdown (n = 5)

Figure S3. Association between LAMA4 expression and metastasis. (A) qRT-PCR analysis of LAMA4 mRNA levels in WT and LAMA4-depleted pancreatic cancer cell lines. Samples were normalized against GAPDH mRNA levels. (B) The effects of LAMA4 knockdown on cell viability was measured in AsPC1 cells. (C) The effects of LAMA4 knockdown on cell migration and invasion were examined in AsPC1 cells. LAMA4 knockdown did not affect cell migration and invasion in vitro. (D) *In vivo* IVIS images of tumor growth of luciferase-expressing AsPC-1 cells (WT or LAMA4-depleted) implanted in spleen of mice after the indicated times. Tumor tissues on liver were recognized as white nodules on the periphery of liver. Quantitative comparison of signals from the IVIS luciferase images was performed. (E) Tumors liver colonization condition on day 28 of IVIS examination. The tumors on liver were recognized as white nodules on liver. (F) IHC staining confirming successful downregulation of LAMA4 in tumor tissues in livers. Quantification of LAMA4 staining was carried out for comparison.

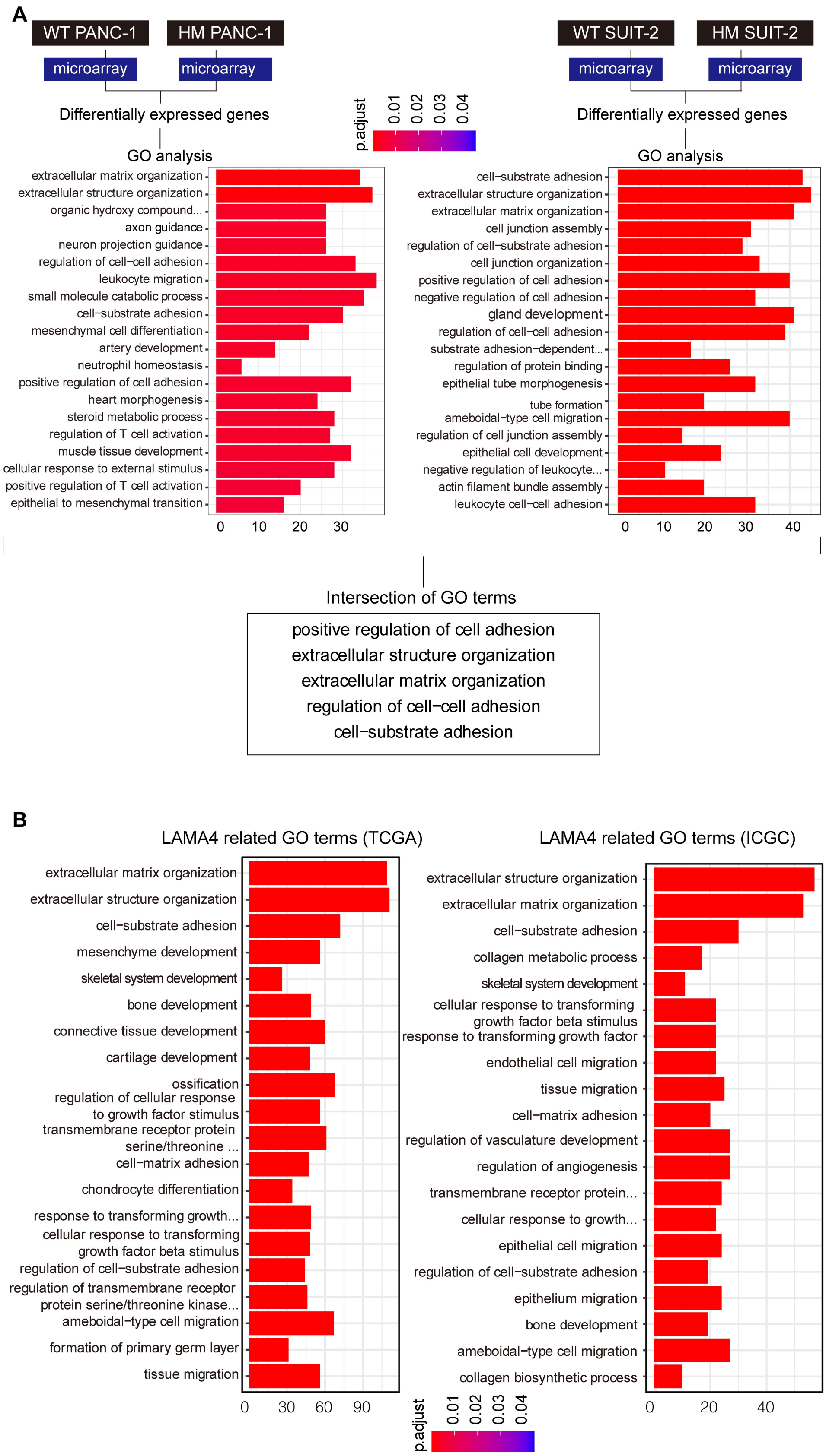
A
WT PANC-1
HM PANC-1
microarray
microarray
Differentially expressed genes
GO analysis
p.adjust
0.01
0.02
0.03
0.04
extracellular matrix organization
extracellular structure organization
organic hydroxy compound...
axon guidance
neuron projection guidance
regulation of cell−cell adhesion
leukocyte migration
small molecule catabolic process
cell−substrate adhesion
mesenchymal cell differentiation
artery development
neutrophil homeostasis
positive regulation of cell adhesion
heart morphogenesis
steroid metabolic process
regulation of T cell activation
muscle tissue development
cellular response to external stimulus
positive regulation of T cell activation
epithelial to mesenchymal transition
0
10
20
30
WT SUIT-2
HM SUIT-2
microarray
microarray
Differentially expressed genes
GO analysis
cell−substrate adhesion
extracellular structure organization
extracellular matrix organization
cell junction assembly
regulation of cell−substrate adhesion
cell junction organization
positive regulation of cell adhesion
negative regulation of cell adhesion
gland development
regulation of cell−cell adhesion
substrate adhesion−dependent...
regulation of protein binding
epithelial tube morphogenesis
tube formation
ameboidal−type cell migration
regulation of cell junction assembly
epithelial cell development
negative regulation of leukocyte...
actin filament bundle assembly
leukocyte cell−cell adhesion
0
10
20
30
40
Intersection of GO terms
positive regulation of cell adhesion
extracellular structure organization
extracellular matrix organization
regulation of cell−cell adhesion
cell−substrate adhesion
B
LAMA4 related GO terms (TCGA)
extracellular matrix organization
extracellular structure organization
cell−substrate adhesion
mesenchyme development
skeletal system development
bone development
connective tissue development
cartilage development
ossification
regulation of cellular response to growth factor stimulus
transmembrane receptor protein serine/threonine ...
cell−matrix adhesion
chondrocyte differentiation
response to transforming growth...
cellular response to transforming growth factor beta stimulus
regulation of cell−substrate adhesion
regulation of transmembrane receptor protein serine/threonine kinase...
ameboidal−type cell migration
formation of primary germ layer
tissue migration
0
30
60
90
LAMA4 related GO terms (ICGC)
extracellular structure organization
extracellular matrix organization
cell−substrate adhesion
collagen metabolic process
skeletal system development
cellular response to transforming growth factor beta stimulus
response to transforming growth factor
endothelial cell migration
tissue migration
cell−matrix adhesion
regulation of vasculature development
regulation of angiogenesis
transmembrane receptor protein...
cellular response to growth...
epithelial cell migration
regulation of cell−substrate adhesion
epithelium migration
bone development
ameboidal−type cell migration
collagen biosynthetic process
0
20
40
p.adjust
0.01
0.02
0.03
0.04

Figure S4. Functional annotation of LAMA4. (A) GO enrichment analysis of differentially expressed genes between WT and HM pancreatic cancer cell lines. Gene expression profiling data of WT and HM pancreatic cancer cell lines were analyzed and differentially expressed genes were identified ($|logFC| > 1$, $p < 0.05$). The packages used for R program were "clusterProfiler", "org.Hs.eg.db", "enrichplot" and "ggplot2". The setup of parameters for r script were pvalueCutoff =0.05, qvalueCutoff = 0.05. (B) LAMA4-related gene ontology terms. The genes that strongly correlated with LAMA4 were screened by Spearman's correlation analysis (spearman $|R| > 0.4$) based on the TCGA and ICGC datasets. Biofunctions of the genes were explored by GO analysis.

R script

1. R script for GO analysis:

```
library("clusterProfiler")
library("org.Hs.eg.db")
library("enrichplot")
library("ggplot2")
term <- enrichGO(gene = gene,
            OrgDb = org.Hs.eg.db,
            pvalueCutoff =0.05,
            qvalueCutoff = 0.05,
            ont="all",
            readable =T)
```

2. R script for violin-boxplot wilcox analysis:

```
library(ggplot2)
library(ggpubr)
Sys.setenv(LANGUAGE = "en")
options(stringsAsFactors = FALSE)
tmp <- read.csv("input.csv", row.names = NULL, check.names = F, header = T,
stringsAsFactors = F)
head(tmp)
table(tmp$type)
p <- wilcox.test(tmp[which(tmp$type == ""),""],tmp[which(tmp$type == ""),""])$p.value
```

3. R script for survival analysis:

```
library(survival)
library(survminer)
svdata <- read.csv ("input.csv", header = T, row.names = 1)
dim(svdata)
res.cut <- surv_cutpoint(svdata, time = "futime",
                event = "fustat",
                variables = names(svdata)[3:ncol(svdata)],
                minprop = 0.3)
res.cat <- surv_categorize(res.cut)
my.surv <- Surv(res.cat$futime, res.cat$fustat)
pl<-list()
```

```
for (i in colnames(res.cat)[3:ncol(svdata)]) {
  group <- res.cat[,i]
  survival_dat <- data.frame(group = group)
  fit <- survfit(my.surv ~ group)
```
